# Viral Haplotypes in COVID-19 Patients Associated With Prolonged Viral Shedding

**DOI:** 10.3389/fcimb.2021.715143

**Published:** 2021-11-03

**Authors:** Yingping Wu, Shufa Zheng, Tian Liu, Xueke Liu, Huina Tang, Yutong He, Wei Xu, Lele Li, Wenxu Yu, Ke Xing, Xiaoping Xia

**Affiliations:** ^1^ Fourth Affiliated Hospital, College of Medicine, Zhejiang University, Jinhua, China; ^2^ State Key Laboratory for Diagnosis and Treatment of Infectious Diseases, National Clinical Research Centre for Infectious Diseases, Collaborative Innovation Centre for Diagnosis and Treatment of Infectious Diseases, First Affiliated Hospital, College of Medicine, Zhejiang University, Hangzhou, China; ^3^ School of Life Sciences, Guangzhou University, Guangzhou, China

**Keywords:** SARS-CoV-2, whole-genome sequencing, viral haplotype, COVID-19, prolonged viral shedding

## Abstract

**Background:**

Recently, more patients who recovered from the novel coronavirus disease 2019 (COVID-19) may later test positive for severe acute respiratory syndrome coronavirus 2 (SARS-CoV-2) again using reverse transcription-polymerase chain reaction (RT-PCR) testing. Even though it is still controversial about the possible explanation for clinical cases of long-term viral shedding, it remains unclear whether the persistent viral shedding means re-infection or recurrence.

**Methods:**

Specimens were collected from three COVID-19-confirmed patients, and whole-genome sequencing was performed on these clinical specimens during their first hospital admission with a high viral load of SARS-CoV-2. Laboratory tests were examined and analyzed throughout the whole course of the disease. Phylogenetic analysis was carried out for SARS-CoV-2 haplotypes.

**Results:**

We found haplotypes of SARS-CoV-2 co-infection in two COVID-19 patients (YW01 and YW03) with a long period of hospitalization. However, only one haplotype was observed in the other patient with chronic lymphocytic leukemia (YW02), which was verified as one kind of viral haplotype. Patients YW01 and YW02 were admitted to the hospital after being infected with COVID-19 as members of a family cluster, but they had different haplotype characteristics in the early stage of infection; YW01 and YW03 were from different infection sources; however, similar haplotypes were found together.

**Conclusion:**

These findings show that haplotype diversity of SARS-CoV-2 may result in viral adaptation for persistent shedding in multiple recurrences of COVID-19 patients, who met the discharge requirement. However, the correlation between haplotype diversity of SARS-CoV-2 virus and immune status is not absolute. It showed important implications for the clinical management strategies for COVID-19 patients with long-term hospitalization or cases of recurrence.

## Introduction

The COVID-19 pandemic caused by the SARS-CoV-2 virus, was first reported from Wuhan’s Huanan Seafood Market in China in December 2019 (Li et al., 2020a; Zhu et al., 2020). Since the early cases were from China, further evidences pointed the outbreak of the SARS-CoV-2 virus to a zoonotic origin of the epidemic (Zhou et al., 2020). Some studies also found that an early COVID-19 pandemic had spread among asymptomatic individuals in other countries several months before the first case was identified in China (Apolone et al., 2020; Zehender et al., 2020). However, previous studies have revealed that other viruses could contribute to the cross-reactivity of SARS-CoV-2 antibodies (Ng et al., 2020). It is well known that SARS-CoV-2 is an infectious respiratory virus, and is highly contagious with a low-mortality rate (Wu et al., 2020a), so tracing early COVID-19 cases associated with Wuhan could contribute to revealing the molecular characterization of SARS-CoV-2.

Compared with severely affected COVID-19 patients, it has been reported that mild asymptomatic infections and moderate infections with lung imaging manifestations are the main clinical types in China’s new COVID-19 Pneumonia Diagnosis and Treatment Guidelines (6th Edition) (Ma et al., 2020). Previous reports showed that the above two infectious groups had better recovery with fewer hospitalization days (Zheng et al., 2020). However, based on the criterion of 2 consecutive negative PCR test results, a high recovery rate of 16.8% (An et al., 2020) or 21.4% (Xiao et al., 2020) has been found from COVID-19 patients who had positive PCR test results. In our previous study, a recovery rate of 31% has been reported (Li et al., 2020b). Studies have shown that the detection methodology and intermittent shedding of SARS-CoV-2 will have an impact on the clinical laboratory detection of nucleic acid (Li et al., 2020c). Five cases of re-infection have been reported (To et al., 2020; Tillett et al., 2021; Bongiovanni, 2021). However, there are still some controversies against the persistent viral shedding of re-infection or recurrence, and those conclusion are key to rule out the disease control strategies and clinical treatment.

In this study, we conducted next-generation sequencing on respiratory samples from three patients who were confirmed as clinical moderate COVID-19 disease, and had long hospitalization stays in Yiwu, China. They were distinctly characterized by the two familial connections of the disease. Through the analysis of viral genome sequencing and clinical follow-up of the three patients, we found that the cause of prolonged viral shedding may be due to the existence of viral quasi-species during their first and second hospitalizations. These findings suggest that we should pay attention to the identification of SARS-CoV-2 haplotypes, which is advantageous to formulate more accurate discharge criteria, reduce “recovery” cases by confirming whether viral haplotypes existed, and avoid the possibility of further human-to-human transmission.

## Materials and Methods

### Patients and Samples

The three patients (named YW01, YW02, and YW03) in this study had been hospitalized with an acute respiratory illness (pneumonia) in the Fourth Affiliated Hospital Zhejiang University School of Medicine in Zhejiang Province of China, a hospital designated for the diagnosis and treatment of COVID-19 patients in February 2020, showing lung involvement with ground-glass opacity. They had an onset of symptoms in January 2020 and had been diagnosed as moderate cases of COVID-19 according to the severity of clinical symptoms. However, they were readmitted in March 2020, after being discharged in late January 2020 based on the COVID-19 Pneumonia Diagnosis and Treatment Guidelines in China. Respiratory tract specimens, such as nasopharyngeal swabs and sputum, and peripheral blood in our hospital and these biological samples were confirmed as being positive for SARS-CoV-2 nucleic acids. This study was approved by the Ethics Committee of the Fourth Affiliated Hospital, College of Medicine, Zhejiang University (Approval No. K20200026). Written informed consent was obtained from all patients when samples were collected. Patients were informed about the surveillance before providing written consents, and data directly related to disease control were collected and anonymized for analysis.

### Laboratory Tests and Computed Tomography Scanning

Follow-up observation and monitoring indicators were performed during hospitalization. Severe acute respiratory syndrome coronavirus (SARS-CoV-2) ribonucleic acid (RNA) was tested using real-time reverse transcription-polymerase chain reaction (RT-PCR) kits (Liferiver, Shanghai, China). Immunoglobulin G (IgG) and Immunoglobulin M (IgM) tests were performed against SARS-CoV-2 nucleoproteins using colloidal gold immunodot assay (Innovita, Beijing, China). Whole blood analyses were performed with an automated hematology analyzer (Sysmex, Kobe, Japan) and cytokines were detected by a flow cytometry assay (Agilent, California, USA). Latex-enhanced immunoturbidimetry was used for C-reactive protein examinations (Mindray, Shenzhen, China). CT scanning was performed routinely as a follow-up after discharge.

### Whole Genome Sequencing of SARS-CoV-2

SARS-CoV-2 RNA was extracted from 200 µl of respiratory tract specimens from three COVID-19 patients with a high viral load at early stage of first hospitalization, using QIAamp Viral RNA Mini Kit (QIAGEN, Hilden, Germany) according to the manufacturer’s instructions. Based on the crossing point (Cp) value of < 25 and 25-32 to assess the viral load, a different nucleic acid pretreatment was performed using probe capture technology to remove the anthropogenic nucleic acid. Qualified libraries were measured and checked by the Invitrogen Qubit 2.0 fluorometer (ThermoFisher, Foster City, CA, USA) and Agilent 2100 Bioanalyzer (Agilent, California, USA). Then, virus genomes were sequenced with PE150 using the NovaSeq 6000 platform (Illumina, San Diego, CA, USA) before bioinformatics analysis including data filtering and genome assembly. Genome sequences of Patients YW01, YW02, and YW03 described in this manuscript (Accession ID: EPI_ISL_3501737, EPI_ISL_3501738, EPI_ISL_3501739, EPI_ISL_3501740, EPI_ISL_3501741), are available from GISAID (https://www.gisaid.org/).

### Alignment With Viral Genome and Variants Calling

The sequence reads were aligned to the SARS-CoV-2 reference strain Wuhan-Hu-1 (NCBI accession NC_045512.2) using BWA v0.7.17 (Li and Durbin, 2009). The SAM files were subsequently processed by SAM tools, and duplicates were removed by Picard v2.7.1 (http://broadinstitute.github.io/picard/). Variants were called with LoFreq variants caller (Wilm et al., 2012), requiring both sequencing quality and MAPQ to be≥30, and subsequently confirmed by V-pipe v2.0 (Posada-Céspedes et al., 2021). All variants were annotated by SnpEff (Cingolani et al., 2012). The sequencing depth was analyzed and plotted in deepTools v3.5.0 (Cingolani et al., 2012).

### Haplotype Reconstruction

Haplotype reconstructions were performed by a maximum-likelihood framework aBayesQR v 1.0.0 (Ahn and Vikalo, 2018), with the SNV_thres set to 0.05, and only haplotypes with frequency ≥ 0.10 were included in the following analysis.

### GISAID Genome Sequences and Annotation

A total of 350 SARS-CoV-2 genome sequences, which belong to the GISAID S clade from January to February 2020, were downloaded from GISAID (https://www.gisaid.org/) (Elbe and Buckland-Merrett, 2017). By removing sequences with gaps (Ns) and ambiguous characters, 296 genome sequences from GISAID were finally included in our analysis. All these genome sequences were annotated using CDSs annotated in NC_045512 by Exonerate (–model protein2genome: bestfit –score 5 -g y) (Slater and Birney, 2005; Abascal et al., 2010).

### Phylogenetic Analysis and Haplotype Network Analysis

Codon-based multiple sequence alignments were performed using Perl script translatorx_vLocal.pl (Abascal et al., 2010) and MAFFT v7.310 (Katoh and Standley, 2013). Maximum likelihood phylogenetic analysis based on all coding regions was conducted by RAxML v8.2.12 (Stamatakis, 2014), with 1000 repetitions performed *via* bootstrapping. The best-fit nucleotide substitution model (GTR+G) was determined using the Akaike information criterion (AIC), as implemented in jModelTest v2.1.7 (Darriba et al., 2012). Phylogenetic trees were visualized in iTOL (Letunic and Bork, 2019), identical sequences were collapsed. The haplotype data were generated in DnaSP v6.12.03 (Rozas et al., 2017), and a median-joining network was constructed by PopART v1.7 (Leigh and Bryant, 2015).

### Linkage Disequilibrium Analysis

The linkage disequilibrium of the specific sites was analyzed and visualized by Haploview v4.2 (Barrett et al., 2005).

### Evolutionary Analysis

The evolutionary analysis was performed in the Datamonkey Adaptive Evolution Server (https://www.datamonkey.org/) (Kosakovsky Pond et al., 2020) with the adaptive Branch-Site Random Effects Likelihood (*aBSREL*) method (Smith et al., 2015) and also by CodeML with a branch model (Yang, 2007).

### Structural Modeling

Protein 3D structures were modeled by the SWISS-MODEL server (https://swissmodel.expasy.org/) (Waterhouse et al., 2018) and visualized in PyMOL (the PyMOL Molecular Graphics System, Version 1.2r3pre, Schrödinger, LLC.). Similarities between structures were evaluated by MATRAS: MArkovian TRAnsition of Structure evolution (http://strcomp.protein.osaka-u.ac.jp/matras/) (Kawabata and Nishikawa, 2000). *R_dis_* is normalized distance score (Sdis), defined as $Rdis\;(\text{A},\text{B})=100\times \frac{\text{Sdis}(\text{A},\text{B})-\text{Smin}}{\text{Smax}-\text{Smin}}$ , where Sdis (A,B) is a raw distance score between proteins A and B, and Smax and Smin is the maximum and minimum value of the score correspondingly. DRMS is the root mean square deviation (angstrom) of distances between Cbeta atom positions of aligned residues

### Statistical Analysis

Statistical analysis was performed using SPSS Statistics version 23 (IBM). A P- value of ≤ 0.05 was considered significant.

## Results

### Case Report

The three cases were second-generation COVID-19 infections from Wuhan. YW01 and YW02 belong to the same familial cluster because both were at the same family party together on January 12, 2020. Before YW01 was exposed to the infection, YW02 had been exposed to a common Wuhan-related first-generation of COVID-19 patient, who was the son of YW02, on January 17, 2020. However, Patient YW03 was associated with another familial cluster infection. All of them had a long follow-up of 76, 69, and 74 days, respectively.

**Figure 1:**
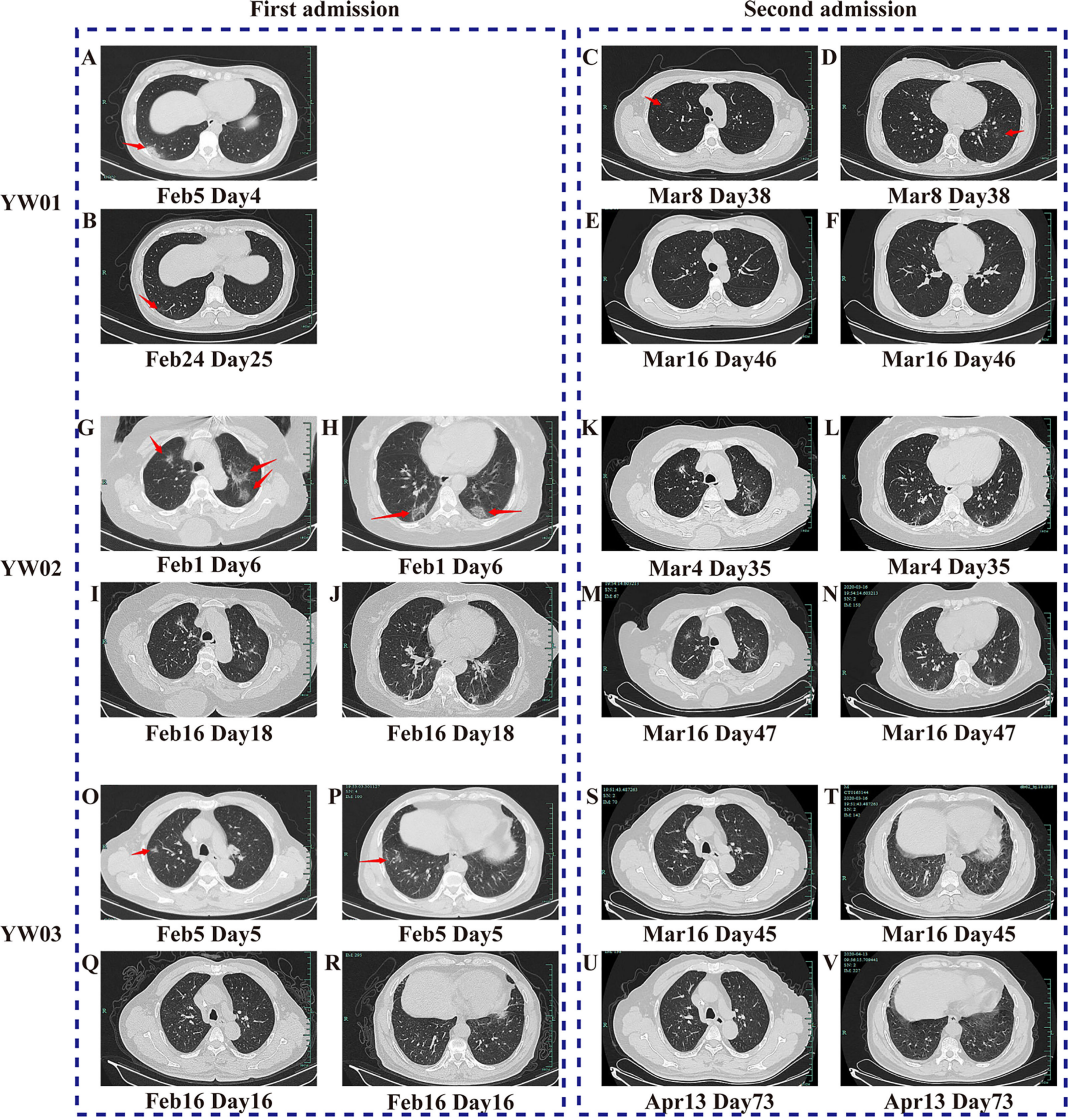
CT scanning images of the three COVID-19 patients during two hospitalizations. The images of **(A–F)** belong to Patient YW01, **(G–N)** belong to YW02, and **(O-V)** belong to YW03. They showed the typical imaging changes of COVID-19 during twice episodes, from density-increased patchy consolidation and ground-glass shadow to absorption of strip high-density shadows of the lung. The red arrows in **(A–D, G, H, O, P)** indicate typical CT scan signs of COVID-19.

Patient YW01 was a 32-year-old healthy woman who was diagnosed with COVID-19 with a history of familial clusters in our hospital (Yu et al., 2020). Her chest X-ray was normal upon admission. Because she had been breastfeeding, she was treated with atomized inhalation of recombinant human interferon α-2b (IFN α-2b) as the antiviral treatment, supplemented with traditional Chinese medicine including Scutellaria, Reed Root, Patchouli, et al. On Day 4, a chest CT scan revealed a dense patchy consolidation and ground-glass opacity in the right lower lobe (**Figure 1A**). Nasopharyngeal swab specimens were positive for SARS-CoV-2 RNA repeatedly, although there was no significant increase in CRP. Subsequently, chest CT scans showed the gradual clearing of the lungs. On Day 28, the patient was discharged as three consecutive nasopharyngeal swabs tested negative for SARS-CoV-2 RNA, and the CT scan showed obvious absorption (**Figure 1B**).

However, YW01 came to our hospital for her follow-up 8 days after being discharged on Day 37. Her chest CT findings showed that her two lungs had new ground-glass opacity (**Figures 1C, D**), while the original lesion in the right lower lobe was basically absorbed and the SARS-CoV-2 RNA test was positive again. After ten days of the second hospitalization, YW01 was discharged again and the course of the disease persisted for 48 days until CT results showed progressive absorption of the lesion (**Figures 1E, F**).

A 72-year-old woman with a history of hypertension, named YW02, was diagnosed with COVID-19 and chronic lymphocytic leukemia (CLL) (Ye et al., 2020), whose chest CT showed multiple areas of ground-glass opacities under the pleura of both lungs and tested positive in a SARS CoV-2 RNA test (**Figures 1G, H**). She received a modified combination therapy consisting of antiviral treatment, Thymosin, as well as intravenous immunoglobulin (IVIG), supplemented with Chinese herbal medicine as adjuvant therapy similar to Patient YW01, and she was discharged when CT results showed absorption (**Figures 1I, J**). During the long period of follow- up, her chest CT showed a transient progression of the lesion followed by gradual absorption (**Figures 1K, L**). The patient was readmitted to the hospital for isolation, because she tested positive in the SARS CoV-2 RNA test on Day 35 and Day 64 of the overall course; after treatment, the virus test showed negative and the patient was discharged on Day 52 and on Day 69, respectively. However, the last CT scan on Day 47 showed an improvement (**Figures 1M, N**).

Patient YW03 was a previously healthy 69-year-old man with a clear epidemiological history and positive result for the SARS-CoV-2 RNA test. His chest X-ray was normal upon admission. Patient YW03 received antiviral therapy (Lopinavir/ritonavir, Arbidol, aerosol inhalation of Interferon α-2b), Thymosin, and Chinese herbs similarly. Five days later, a chest CT scan showed a patchy ground-glass opacity in the upper and lower lobes of the right lung (**Figures 1O, P**). After treatment, a chest CT showed obvious absorption of inflammation in both lungs on Day 16, although a respiratory specimen repeated positive in a nucleic acid test (**Figures 1Q, R**). On Day 29, he was discharged since respiratory specimens were consecutively negative in a nucleic acid test. Thirteen days after his first discharge, the patient was readmitted because of consecutively positive results for SARS-CoV-2 during follow-up on Day 43 and was provided similar treatment as he had received previously. IVIG and Hydroxychloroquine were used as a try. His respiratory specimens were continuously positive in a nucleic acid test, though a chest CT showed that ground- glass opacity was basically absorbed (**Figures 1S, T**). Patient YW03 was discharged again 18 days later. In about 2 weeks after Day 73, a follow-up chest CT scan showed no sign of recurrence (**Figures 1U, V**) and the SARS-CoV-2 RNA test was negative.

### Epidemiological and Clinical Characteristics of 3 Viral Recurrence Patients

**Figure 2:**
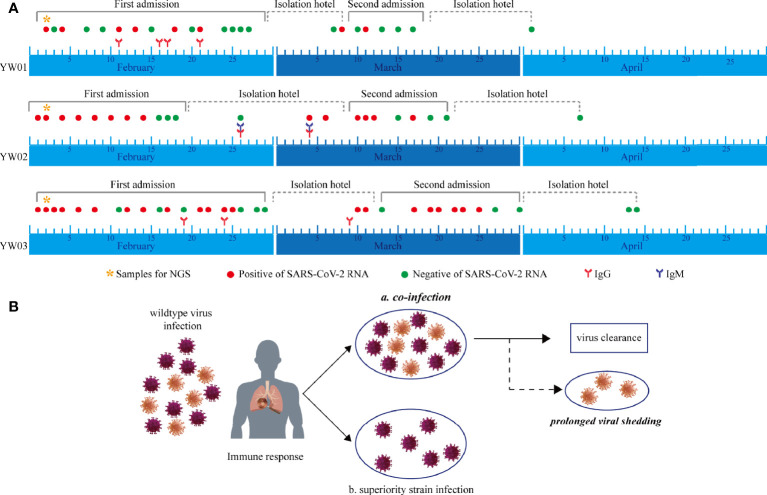
Timeline during the whole course of COVID-19, including first and second episodes. **(A)** Serial SARS-CoV-2 RNA testing, anti-SARS-CoV-2 IgM/IgG, and specimens for NGS were performed. CT, computed tomography; RNA, ribonucleic acid; NGS, next-generation sequencing; IgM, immunoglobulin M; IgG, immunoglobulin G; SARS-CoV-2; severe acute respiratory syndrome coronavirus 2; COVID-19, coronavirus disease 2019. **(B)** Hypothesis of haplotype or quasi-species co-infection of SARS-CoV-2.

The clinical diagnosis timeline of the three patients with confirmed COVID-19 is shown in **Figure 2A**. The serum specimens for anti-SARS-CoV-2 antibodies (IgM and IgG) tests were collected during the first admission and isolation hotel period, while sputum or nasal swabs for SARS-CoV-2 RNA testing were sampled throughout the whole course of the disease. The continuous SARS-CoV-2 RNA positive results showed the prolonged viral shedding, while anti-SARS-CoV-2 IgG antibodies were detected in the serum of the patients in February.

Previous studies had demonstrated the need for monitoring kinetic changes of inflammatory cytokine levels in COVID-19 patients (Liu et al., 2020). Therefore, we continuously monitored the inflammatory levels during repeat admissions, such as interleukin (IL) -2, IL-4, IL-6, IL-10, IFN-γ, and TNF-α, in the serum of the three long hospitalization patient cohort (**Figure S1**). All patients had moderate symptoms of COVID-19 with a long viral shedding period in our study. It was considered that there was little significant difference in the serum levels of these cytokines in mild patients in previous studies, however, our study showed the contrary. Except for IL-6, examined cytokines in Patients YW01 and YW03 showed higher levels during the first admission. While Patients YW01 and YW03 had high values of IL-2, IL-4, IL-6, and IFN-γ in the second admission. However, the IL-6 levels in all of the three patients exceeded the upper limit of normal value (6.61 pg/ml) in the second admission. The only significant difference in serum TNF-α level was observed in Patients YW01 and YW03 between the first and second admissions. Moreover, we observed higher IL-2, IL-4, and IFN-γ levels in Patient YW01 than Patients YW02 and YW03 in the second admission; however, the differences were not significant in the first admission.

### Variants Landscape and Haplotypes Reconstruction

**Figure 3:**
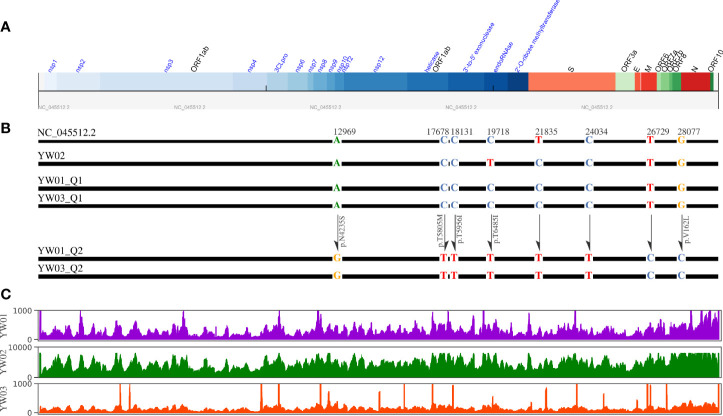
Diagram of YW SARS-CoV-2 haplotypes. **(A)** Genome structure of SARS-CoV-2 reference strain (NC_045512.2); **(B)** Schematic diagram of YW haplotypes, where only the 8 specific variants that detected in different haplotypes simultaneously are plotted; **(C)** Sequencing coverage of YW samples.

The sequencing reads coverage was shown in **Figure 3C**, the average sequencing depth of YW01, YW02 and YW03 were 809.7, 5343.7, and 441.3, respectively. By aligning to the NCBI reference strain Wuhan-Hu-1 (Accession NC_045512.2), our strains showed more than 99.9% identity with the reference genome. A total of 37 variants (allele frequency, AF>5%) were detected in our YW SARS-CoV-2 strains, including 22 missense, 14 synonymous and 1 stop gained variants (**Table 1**). Co-occurrence of substitutions C8782T and T28144C suggested all our strains belonged to the GISAID S clade. The number of variants per sample showed no correlation with the sequencing depth (*R*= -0.96, *p* = 0.18).

**Table 1:** Variants in genome.

Position	Ref	Alt	DP.YW01	Af.YW01	DP.YW02	Af.YW02	DP.YW03	Af.YW03	Alt Frequency in GISAID	Gene	Mutation type	Protein Change	Impact
**1963**	T	C	167	0.065868	3278	0	97	0	0.00E+00	Orf1ab	Synonymous	p.Ala566Ala	
**3055**	T	G	109	0	2064	0	41	0.097561	0.00E+00	Orf1ab	Missense	p.Asp930Glu	MODERATE
**3076**	A	G	111	0	2202	0	42	0.142857	1.06E-05	Orf1ab	Synonymous	p.Glu937Glu	LOW
**4038**	A	C	337	0	4885	0.062027	112	0	0.00E+00	Orf1ab	Missense	p.Asp1258Ala	
**4887**	C	A	266	0	4582	0	141	0.056738	0.00E+00	Orf1ab	Missense	p.Pro1541His	MODERATE
8782*	C	T	174	1	3027	0.998679	109	0.981651	2.82E-02	Orf1ab	Synonymous	p.Ser2839Ser	LOW
**9764**	T	G	240	0	3385	0	735	0.087075	0.00E+00	Orf1ab	Missense	p.Phe3167Val	MODERATE
**9770**	G	T	240	0	2945	0	732	0.101093	0.00E+00	Orf1ab	Missense	p.Gly3169Cys	MODERATE
**12969#**	A	G	260	0.115385	4948	0	168	0.22619	5.23E-06	Orf1ab	Missense	p.Asn4235Ser	MODERATE
**13255**	C	T	195	0.066667	3190	0	111	0.189189	5.23E-06	Orf1ab	Synonymous	p.Cys4330Cys	LOW
**14831**	G	C	317	0.501577	3825	0	71	0	0.00E+00	Orf1ab	Missense	p.Cys4856Ser	MODERATE
**15909**	T	C	360	0	4247	0	113	0.079646	1.60E-05	Orf1ab	Synonymous	p.Gly5215Gly	LOW
**17678#**	C	T	426	0.103286	7222	0	209	0.186603	1.63E-03	Orf1ab	Missense	p.Thr5805Met	MODERATE
**18131#**	C	T	443	0.060948	5196	0	192	0.286458	6.87E-04	Orf1ab	Missense	p.Thr5956Ile	MODERATE
**19132**	G	T	584	0.207192	6875	0.104291	158	0	0.00E+00	Orf1ab	Stop gained	p.Glu6290*	HIGH
**19137**	A	C	580	0.177586	6930	0.08961	159	0	0.00E+00	Orf1ab	Missense	p.Leu6291Phe	MODERATE
**19166**	A	G	100	0.29	2899	0	44	0	1.60E-05	Orf1ab	Missense	p.Lys6301Arg	MODERATE
**19718#**	C	T	332	0.096386	5367	0.994038	159	0.119497	1.84E-02	Orf1ab	Missense	p.Thr6485Ile	MODERATE
**21796**	G	A	179	0	3025	0	86	0.348837	5.23E-06	S	Synonymous	p.Arg78Arg	LOW
**21835#**	T	C	143	0.216783	2347	0.988496	87	0.149425	1.60E-05	S	Synonymous	p.Tyr91Tyr	LOW
**21861**	T	C	155	0	2817	0	89	0.089888	5.86E-05	S	Missense	p.Ile100Thr	MODERATE
**22114**	T	C	124	0	2950	0	65	0.076923	1.06E-05	S	Synonymous	p.Gly184Gly	LOW
**22506**	C	A	214	0	4793	0.074275	142	0	0.00E+00	S	Missense	p.Thr315Asn	MODERATE
**22990**	T	C	350	0	5606	0	137	0.051095	5.23E-06	S	Synonymous	p.Gly476Gly	LOW
**23308**	T	G	391	0	6402	0	158	0.056962	0.00E+00	S	Synonymous	p.Leu582Leu	LOW
**24034#**	C	T	254	0.090551	4499	0	134	0.238806	3.19E-03	S	Synonymous	p.Asn824Asn	LOW
**24942**	A	T	434	0	6105	0.058968	205	0	1.06E-05	S	Missense	p.Asp1127Val	MODERATE
**25073**	G	A	250	0.108	4656	0	102	0	2.12E-05	S	Missense	p.Gly1171Ser	MODERATE
**25156**	C	T	401	0.316708	3671	0	87	0	1.01E-04	S	Synonymous	p.Ile1198Ile	LOW
**25160**	C	T	429	0.286713	3777	0	89	0	1.01E-04	S	Missense	p.Leu1200Phe	MODERATE
**25952**	G	T	97	0	1874	0	51	0.313726	0.00E+00	ORF3a	Missense	p.Gly187Val	MODERATE
**26262**	G	T	297	0.080808	5670	0	144	0.111111	1.60E-04	E	Synonymous	p.Ser6Ser	LOW
**26324**	T	G	276	0	5081	0	396	0.643939	0.00E+00	E	Missense	p.Leu27Trp	MODERATE
**26729#**	T	C	316	0.085443	5402	0	131	0.305344	1.96E-03	M	Synonymous	p.Ala69Ala	LOW
**27987**	G	C	419	0	7494	0	213	0.352113	5.23E-06	ORF8	Missense	p.Val32Leu	MODERATE
**28077#**	G	C	333	0.057057	5396	0	150	0.313333	2.16E-03	ORF8	Missense	p.Val62Leu	MODERATE
28144*	T	C	433	0.993072	6690	0.997309	182	1	2.82E-02	ORF8	Missense	p.Leu84Ser	MODERATE

*: Mutation C8782T and T28144C indicate all the three strains are belong to GISAID S clade; #: mutations in blue are observed simultaneously in YW01 and YW03.

Interestingly, 8 specific variants with relatively low allele frequencies (range from 5~31%) were detected simultaneously in YW01 and YW03 samples, including A12696G, C7678T, C18131T, C19718T, T21835C, C24034T, T26729C, and G28077C (**Table 1**). This similar intra-host variants spectrum could be explained by transmission of quasi-species (QS) between patients or co-infection of different SARS-CoV-2 strains (**Figure 2B**).

Considering low level intra-host diversity in SARS-CoV-2 infection, we chose aBayesQR to get whole length haplotypes, which is designed for highly identical haplotype reconstruction. By using a maximum-likelihood framework aBayesQR, we reconstructed viral haplotypes for our samples. With a frequency cutoff at 0.1, two haplotypes were reconstructed in both YW01 and YW03 samples, respectively, and one haplotype was found in YW02. Both YW01 Q1 and YW03 Q1 harbored variant T21835C, whereas haplotype YW01 Q2 and YW03 Q2 harbored variants A12696G, C17678T, C18131T, C19718T, C24034T, T26729C, and G28077C (**Figures 3A, B**), which indicates YW01 Q1 and YW03 Q1 came from one ancestral strain/quasi-species and YW01 Q2 and YW03 Q2 came from another.

**Figure 4:**
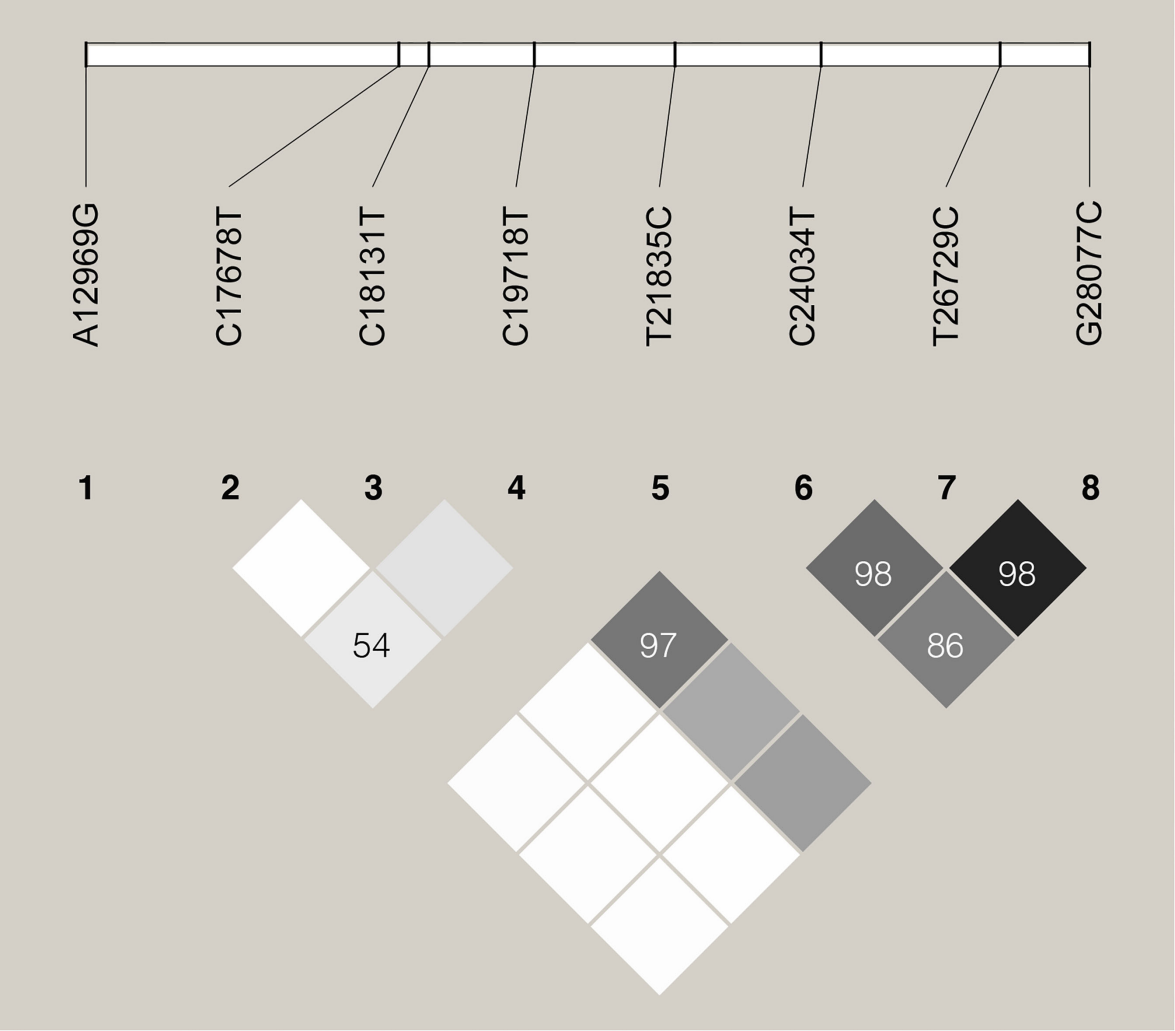
Linkage disequilibrium plot of haplotypes of the 8 specific sites. *D’* values are indicated in percentages within squares.

We further examined the distribution of these 8 specific variants in all the 187,857 SARS-CoV-2 genome sequences deposited in the GISAID Database until Dec. 11^th^, 2020. The allele frequencies of these variants were shown in **Table 1**. Three variants, including C24034T, T26729C, and G28077C, were found highly linked with *D’* larger than 0.85 (**Figure 4**). In addition, the linkage was also observed between C19718T and C24034T with *D’*=0.97.

### Phylogenetic and Haplotype Network Analysis

**Figure 5:**
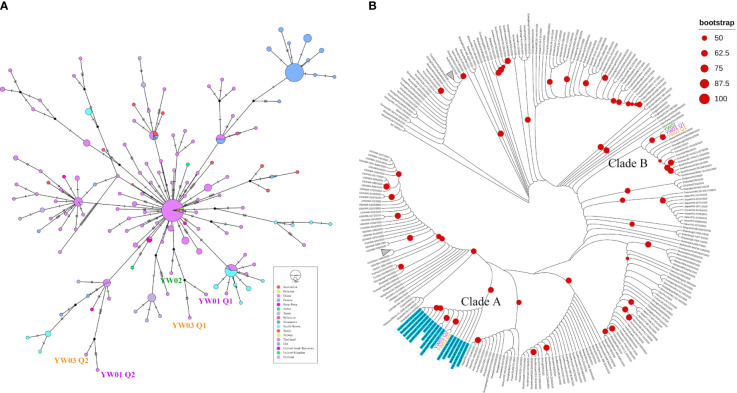
Evolutionary relationship of YW haplotypes and GISAID S strains. **(A)** Median-joining haplotype network of 302 SARS-CoV-2 haplotypes, including 5 YW haplotypes, GISAID S strains from Jan. to Feb. 2020 and NC_045512.2; **(B)** Maximal likelihood phylogenetic tree based on all coding regions of the 302 SARS-CoV-2 showed 5 YW haplotypes were separated into different clades (Clade A and Clade B). Strains harbored variants C24034T, T26729C, and G28077C are highlighted in blue (including YW01 Q2 and YW03 Q2).

As our samples are classified as GISAID S clade, we collected all genome sequences belonging to the S clade from GISAID Database with date from January 2020 to February 2020 to investigate the potential epidemiological linkage between samples. By removing in-complete genome sequences and low-quality genome sequences, a total of 302 SARS-CoV-2 sequences including 5 haplotypes from our YW samples, NC_045512.2 and 296 S clade genome sequences were subjected to haplotype network and phylogenetic analysis. As shown in **Figure 5A**, a median-joining network showed that YW01 Q1, YW03 Q1 and YW02 grouped with the major node from China with 3-6 nucleotide substitutions, whereas YW01 Q2 and YW03 Q2 clustered with South Korea, Vietnam, USA, and some samples from China. In addition, a maximal likelihood phylogenetic analysis based on all coding regions also supported all the 5 YW haplotypes being separated into 2 branches (**Figure 5B**). YW01 Q2 and YW03 Q2 were clustered with 16 SARS-CoV-2 strains which harbored the three-lined variants C24034T, T26729C, and G28077C with a high bootstrap support (Clade A), whereas YW01 Q1, YW02 Q1 and YW02 are found in another clade (Clade B). In line with the results of the haplotype network, these results suggest co-infection of different SARS-CoV-2 strains in our samples.

### Evolutionary Analysis

A few non-synonymous substitutions were observed in ORF1ab of YW01 Q2 and YW03 Q2. We set this branch as the foreground branch and use HyPhy and CodeML to find whether this branch is evolving at faster rates in the ORF1ab coding region than the expectation from the rest strains. Unfortunately, no strong episodic diversifying selection was observed on this branch with ω>1 and *p* value >0.05 by the aBSREL method of HyPhy and CodeML branch models.

### Structural Modeling of the Protein With Missense Variants

The ternary structure of the protein harboring the 5 missense variants was modeled using the SWISS-MODEL server and compared to the wild type by MATRS (MArkovian TRAnsition of Structure evolution server) subsequently. The structural similarities were quantified by using *R_dis_* and dRMS scores (Kawabata and Nishikawa, 2000). As shown in **Figure S2** **(1-5)**, all the five missense variants have little effects on the ternary structures of corresponding proteins. The *R_dis_* scores range from 0.06 to 0.11A, and the dRMS scores range from 97.9 to 100%.

## Discussion

Currently, one of the topical issues for COVID-19 is the recurrence and positive test result of patients after being discharged from hospitals, although they already had two consecutive negative RNA results before being discharged (Wu et al., 2020b). Previous studies supported the explanation of false-negative results of SARS-CoV-2 PCRs (Xie et al., 2020), intermittent shedding of SARS-CoV-2 (Li et al., 2020c) and reinfection (Bongiovanni, 2020; Larson et al., 2020; To et al., 2020). This study presents a novel perspective of prolonged viral shedding of COVID-19. Respiratory samples from the three patients confirmed as COVID-19 were collected at the early stage of first hospitalization, and the whole-genome sequencing was performed on the above clinical samples at their first admission into the hospital. We analyzed the results of nucleic acid tests, inflammatory chemokines, serum SARS-CoV-2 IgG and IgM, chest CT imaging, and other laboratory tests during the whole course of hospital stays. We found that haplotype or quasi-species co-infection of SARS-CoV-2 could occur at an early stage of infection, with a prolonged viral shedding of COVID-19. Another COVID-19 confirmed patient with chronic lymphoblastic leukemia had a long-term viral shedding period of 69 days, who developed multiple relapses after initial discharge, however, only one haplotype virus was found. As a case reported, immunocompromised individual could show persistent SARS-CoV-2 infection and a long viral shedding (Avanzato et al., 2020). Therefore, a weak humoral immune response in YW02 patient may contribute to a long viral shedding. Above mentioned evidence showed the correlation between haplotype or quasi-species diversity of SARS-CoV-2 virus and recurrence in COVID-19 patients, which could result in co-infection and ineffective clearance of SARS-CoV-2 viruses, not only humoral immune response or other risk factors.

As we all know, virus-host interaction during the infectious stage determines the clinical outcome of viral infection, and this process is complex and dynamic (Teng et al., 2016). The existence of a broad host provides convenience for the cross-species transmission of the coronavirus. It is worth noting that the highly infectious human pathogens of SARS-CoV, MERS-CoV, and SARS-CoV-2, which caused the worldwide pandemic, are β-coronaviruses, and current evidence suggests that their natural hosts are bats (Azhar et al., 2014; Menachery et al., 2015; Zhou et al., 2020). Specifically, the epidemic started in December 2019, and Wuhan was the first city with the COVID-19 outbreak, with the early cases being patients in the country. Because of the emergency and limited medical resources at the early outbreak of COVID*-*19 in Wuhan, however, cases associated with it also exhibit the original infectious features in the evolutionary of the SARS-CoV-2 virus. Therefore, studies on next-generation COVID*-*19 cases associated with Wuhan have implications (Chan et al., 2020). In this study, we report the epidemiological, clinical laboratory test, radiological, host transcriptional RNAs, and viral genome characteristics findings of three moderately affected COVID-19 patients. They did not travel to Wuhan and were local residents in Yiwu, Zhejiang Province, China, respectively from two family clusters, in which additional family members had returned from Wuhan. Our findings are consistent with person-to-person transmission of SARS-CoV-2 in family settings, and the subsequent genome analysis findings between virus-host interaction revealed the explanations on the high presence of SARS-CoV-2 RNA positive recurring after discharge from hospital (Li et al., 2020d).

All of COVID-19 patients were considered as moderate cases in our hospital, which is consistent with a study showing that 81% of of COVID-19 cases in China are mild (Wu and McGoogan, 2020c), Multiple studies showed the general susceptibility of the population and have indicated that risk factors for mortality rate include age, being a male, and pre-existing diseases including hypertension, smoking, coronary heart disease, cardiovascular disease, diabetes, lung diseases and neurological symptoms, among others (Abdel-Mannan et al., 2020; St John and Rathore, 2020). Therefore, a host’s immunity has been considered as a reason for the re-occurrence of positive COVID-19 results. The case report of Patient YW02 in our study showed that with CLL the virus was not effectively cleared so that she was re-admitted twice during the 69-day follow-up (Ye et al., 2020). There were some risk factors existing in Patient YW03, such as advanced age and being male, while Patient YW01 was a young female in healthy condition. However, YW01 and YW03 showed a similar co-infection mode with SARS-CoV-2 strains from clades A and B.

Phylogenetic and haplotype analyses of genetic sequences from these patients were performed, and different quasi-species exist simultaneously in the SARS-CoV-2 virus. In agreement with previous studies (Han et al., 2015), haplotype or quasi-species also exist in the SARS-CoV-2 virus. The evidence in our study showed that co-infections happened before the stage of infection exposure. However, the dominant strain underwent individual screening (**Figure 2B**). The subsequent clinical phenotype could be a single dominant strain retained or coexistent with multiple strains of the SARS-CoV-2 virus. Therefore, we speculate that not only immune deficiency, but also SARS-CoV-2 haplotypes in COVID-19 patients could be associated with prolonged viral shedding, even the recurring diagnosis of SARS-CoV-2 RNA test.

Our study had several limitations yet. Firstly, the speculative conclusion of our study should be validated in larger multiple clinical center cohorts, and whole-genome analysis during the second episode was needed for the effect of the SARS-CoV-2 virus quasi-species. Secondly, since viral characteristics in COVID-19 patients were not available during the early stages of the epidemic, seroconversion of IgM could not be observed in the earliest infected patients until late March. Hence, the samples we collected showed no regularity. Thirdly, prolonged viral shedding means that reinfection or recurrence is always controversial and may be influenced by host immune response to SARS-CoV-2 in COVID-19 patients and low-sensitivity of RT-PCR kits in the early stages of February 2020. However, our study provides a detailed correlation analysis of clinical presentation and viral quasi-species at an early stage of exposure infection.

In conclusion, we found the correlation between the haplotypes of the SARS-CoV-2 virus and prolonged viral shedding in Wuhan-associated cases in early 2020 and pointed out the importance of accurate monitoring in SARS-CoV-2 virus molecular typing, which is of great significance for the clinical management strategy of COVID-19 patients with long hospitalization period or in cases of recurrence.

## Data Availability Statement

The original contributions presented in the study are publicly available. The data presented in the study are deposited in the GISAID (https://www.gisaid.org/) repository, accession number EPI_ISL_3501737, EPI_ISL_3501738, EPI_ISL_3501739, EPI_ISL_3501740, EPI_ISL_3501741.

## Ethics Statement

This study was approved by the Ethics Committee of the Fourth Affiliated Hospital, College of Medicine, Zhejiang University (Approval No.K20200026). The patients/participants provided their written informed consent to participate in this study. Written informed consent was obtained from the individual(s) for the publication of any potentially identifiable images or data included in this article.

## Author Contributions

WY and WX collected data. XL and SZ contributed to statistical analyses. TL and HT analyzed CT images. YH, KX, and YW edited figures and tables. YW edited the manuscript. XX and KX reviewed the manuscript. All authors contributed to the article and approved the submitted version.

## Funding

This work was supported by grants from the Joint Funds of the Zhejiang Provincial Natural Science Foundation of China (No. LBY21H190001 to YW), Guangdong Key Project in “Development of new tools for diagnosis and treatment of Autism” (No. 2018B030335001 to KX), Jinhua Science and Technology Bureau (No. 2020XG-28 to XX), and Department of the Education of Zhejiang Province (No. Y202045827 to YW).

## Conflict of Interest

The authors declare that the research was conducted in the absence of any commercial or financial relationships that could be construed as a potential conflict of interest.

## Publisher’s Note

All claims expressed in this article are solely those of the authors and do not necessarily represent those of their affiliated organizations, or those of the publisher, the editors and the reviewers. Any product that may be evaluated in this article, or claim that may be made by its manufacturer, is not guaranteed or endorsed by the publisher.

## Supplementary Material

Supplementary Figure 1 Kinetic analysis of the serum levels of inflammatory cytokines in COVID-19 patients. The concentrations of IL-2 **(A)**, IL-4 **(B)**, IL-6 **(C)**, IL-10 **(D)**, IFN-γ **(E)**, and TNF-α **(F)** in the serum of COVID-19 patients YW01 (red line), YW02 (grey line), and YW03 (blue line) were analyzed at different time points during two hospital admissions. The dotted green line divides the whole course into first and second episodes.

Supplementary Figure 2 Comparison of the predicted ternary structures for the WT (in green) and the mutant (in blue) of SARS-CoV-2 proteins. The mutated sites were colored in red.

## References

[B1] Abascal F. Zardoya R. Telford M. J. (2010). TranslatorX: Multiple Alignment of Nucleotide Sequences Guided by Amino Acid Translations. Nucleic Acids Res. 38 (Web Server issue), W7–W13. doi: 10.1093/nar/gkq291 20435676 PMC2896173

[B2] Abdel-Mannan O. Eyre M. Löbel U. Bamford A. Eltze C. Hameed B.. (2020). Neurologic and Radiographic Findings Associated With COVID-19 Infection in Children. JAMA Neurol. 77 (11), 1440–1445. doi: 10.1001/jamaneurol.2020.2687 32609336 PMC7330822

[B3] Ahn S. Vikalo H. (2018). Abayesqr: A Bayesian Method for Reconstruction of Viral Populations Characterized by Low Diversity. J. Comput. Biol. 25 (7), 637–648. doi: 10.1089/cmb.2017.0249 29480740

[B4] An J. Liao X. Xiao T. Qian S. Yuan J. Ye H.. (2020). Clinical Characteristics of Recovered COVID-19 Patients With Re-Detectable Positive RNA Test. Ann. Trans. Med. 8 (17), 1084. doi: 10.21037/atm-20-5602 PMC7575971 33145303

[B5] Apolone G. Montomoli E. Manenti A. Boeri M. Sabia F. Hyseni I.. (2020). Unexpected Detection of SARS-CoV-2 Antibodies in the Prepandemic Period in Italy. Tumori 300891620974755. doi: 10.1177/0300891620974755 PMC8529295 33176598

[B6] Avanzato V. A. Matson M. J. Seifert S. N. Pryce R. Williamson B. N. Anzick S. L.. (2020). Case Study: Prolonged Infectious SARS-CoV-2 Shedding From an Asymptomatic Immunocompromised Individual With Cancer. Cell 183 (7), 1901–1912.e9. doi: 10.1016/j.cell.2020.10.049 33248470 PMC7640888

[B7] Azhar E. I. El-Kafrawy S. A. Farraj S. A. Hassan A. M. Al-Saeed M. S. Hashem A. M.. (2014). Evidence for Camel-to-Human Transmission of MERS Coronavirus. N. Engl. J. Med. 370 (26), 2499–2505. doi: 10.1056/NEJMoa1401505 24896817

[B8] Barrett J. C. Fry B. Maller J. Daly M. J. (2005). Haploview: Analysis and Visualization of LD and Haplotype Maps. Bioinformatics 21 (2), 263–265. doi: 10.1093/bioinformatics/bth457 15297300

[B9] Bongiovanni M. (2021). COVID-19 Reinfection in a Healthcare Worker. J. Med. Virol. 93 (7), 4058–4059. doi: 10.1002/jmv.26565 32990954 PMC7537129

[B10] Chan J. F. Yuan S. Kok K. H. To K. K. Chu H. Yang J.. (2020). A Familial Cluster of Pneumonia Associated With the 2019 Novel Coronavirus Indicating Person-to-Person Transmission: A Study of a Family Cluster. Lancet 395 (10223), 514–523. doi: 10.1016/S0140-6736(20)30154-9 31986261 PMC7159286

[B11] Cingolani P. Platts A. Wang L. Coon M. Nguyen T. Wang L.. (2012). A Program for Annotating and Predicting the Effects of Single Nucleotide Polymorphisms, SnpEff: SNPs in the Genome of Drosophila Melanogaster Strain W1118; Iso-2; Iso-3. Fly 6 (2), 80–92. doi: 10.4161/fly.19695 22728672 PMC3679285

[B12] Darriba D. Taboada G. L. Doallo R. Posada D. (2012). Jmodeltest 2: More Models, New Heuristics and Parallel Computing. Nat. Methods 9 (8), 772. doi: 10.1038/nmeth.2109 PMC4594756 22847109

[B13] Elbe S. Buckland-Merrett G. (2017). Data, Disease and Diplomacy: GISAID's Innovative Contribution to Global Health. Global Challenges 1 (1), 33–46. doi: 10.1002/gch2.1018 31565258 PMC6607375

[B14] Han Y. Gong L. Sheng J. Liu F. Li X. H. Chen L.. (2015). Prediction of Virological Response by Pretreatment Hepatitis B Virus Reverse Transcriptase Quasispecies Heterogeneity: The Advantage of Using Next-Generation Sequencing. Clin. Microbiol. Infect: Off. Publ. Eur. Soc. Clin. Microbiol. Infect. Dis. 21 (8), 797.e1–797.e7978. doi: 10.1016/j.cmi.2015.03.021 25882357

[B15] Katoh K. Standley D. M. (2013). MAFFT Multiple Sequence Alignment Software Version 7: Improvements in Performance and Usability. Mol. Biol. Evol. 30 (4), 772–780. doi: 10.1093/molbev/mst010 23329690 PMC3603318

[B16] Kawabata T. Nishikawa K. (2000). Protein Structure Comparison Using the Markov Transition Model of Evolution. Proteins 41 (1), 108–122. doi: 10.1002/1097-0134(20001001)41:1<108::AID-PROT130>3.0.CO;2-S 10944398

[B17] Kosakovsky Pond S. L. Poon A. Velazquez R. Weaver S. Hepler N. L. Murrell B.. (2020). HyPhy 2.5-A Customizable Platform for Evolutionary Hypothesis Testing Using Phylogenies. Mol. Biol. Evol. 37 (1), 295–299. doi: 10.1093/molbev/msz197 31504749 PMC8204705

[B18] Larson D. Brodniak S. L. Voegtly L. J. Cer R. Z. Glang L. A. Malagon F. J.. (2020). A Case of Early Re-Infection With SARS-CoV-2. Clin. Infect. Dis: an Off. Publ. Infect. Dis. Soc. America ciaa1436. doi: 10.1093/cid/ciaa1436

[B19] Leigh J. W. Bryant D. (2015). PopART: Full-Feature Software for Haplotype Network Construction. Methods Ecol. Evol. 6 (9), 1110–1116. doi: 10.1111/2041-210X.12410

[B20] Letunic I. Bork P. (2019). Interactive Tree Of Life (iTOL) V4: Recent Updates and New Developments. Nucleic Acids Res. 47 (W1), W256–W259. doi: 10.1093/nar/gkz239 30931475 PMC6602468

[B21] Li H. Durbin R. (2009). Fast and Accurate Short Read Alignment With Burrows-Wheeler Transform. Bioinformatics 25 (14), 1754–1760. doi: 10.1093/bioinformatics/btp324 19451168 PMC2705234

[B22] Li Q. Guan X. Wu P. Wang X. Zhou L. Tong Y.. (2020a). Early Transmission Dynamics in Wuhan, China, of Novel Coronavirus-Infected Pneumonia. N. Engl. J. Med. 382 (13), 1199–1207. doi: 10.1056/NEJMoa2001316 31995857 PMC7121484

[B23] Li Y. Hu Y. Yu Y. Zhang X. Li B. Wu J.. (2020b). Positive Result of Sars-Cov-2 in Faeces and Sputum From Discharged Patients With COVID-19 in Yiwu, China. J. Med. Virol. 92 (10), 1938–1947. doi: 10.1002/jmv.25905 32311109 PMC7264799

[B24] Li Y. Yao L. Li J. Chen L. Song Y. Cai Z.. (2020c). Stability Issues of RT-PCR Testing of SARS-CoV-2 for Hospitalized Patients Clinically Diagnosed With COVID-19. J. Med. Virol. 92 (7), 903–908. doi: 10.1002/jmv.25786 32219885 PMC7228231

[B25] Li Y. Hu Y. Zhang X. Yu Y. Li B. Wu J.. (2020d). Follow-Up Testing of Viral Nucleic Acid in Discharged Patients With Moderate Type of COVID-19. Zhejiang Da Xue Bao Yi Xue Ban 49 (2), 270–274. doi: 10.3785/j.issn.1008-9292.2020.03.11 PMC8800664 32391676

[B26] Liu J. Li S. Liu J. Liang B. Wang X. Wang H.. (2020). Longitudinal Characteristics of Lymphocyte Responses and Cytokine Profiles in the Peripheral Blood of SARS-CoV-2 Infected Patients. EBioMedicine 55, 102763. doi: 10.1016/j.ebiom.2020.102763 32361250 PMC7165294

[B27] Ma L. L. Li B. H. Jin Y. H. Deng T. Ren X. Q. Zeng X. T. (2020). Developments, Evolution, and Implications of National Diagnostic Criteria for COVID-19 in China. Front. Med. 7, 242. doi: 10.3389/fmed.2020.00242 PMC7243174 32574333

[B28] Menachery V. D. Yount B. L. Jr. Debbink K. Agnihothram S. Gralinski L. E. Plante J. A.. (2015). A SARS-Like Cluster of Circulating Bat Coronaviruses Shows Potential for Human Emergence. Nat. Med. 21 (12), 1508–1513. doi: 10.1038/nm.3985 26552008 PMC4797993

[B29] Ng K. W. Faulkner N. Cornish G. H. Rosa A. Harvey R. Hussain S.. (2020). Preexisting and De Novo Humoral Immunity to SARS-CoV-2 in Humans. Science 370 (6522), 1339–1343. doi: 10.1126/science.abe1107 33159009 PMC7857411

[B30] Posada-Céspedes S. Seifert D. Topolsky I. Jablonski K. P. Metzner K. J. Beerenwinkel N. (2021). V-Pipe: A Computational Pipeline for Assessing Viral Genetic Diversity From High-Throughput Data. Bioinformatics 37 (12), 1673–1680. doi: 10.1093/bioinformatics/btab015 PMC8289377 33471068

[B31] Rozas J. Ferrer-Mata A. Sánchez-DelBarrio J. C. Guirao-Rico S. Librado P. Ramos-Onsins S. E.. (2017). DnaSP 6: DNA Sequence Polymorphism Analysis of Large Data Sets. Mol. Biol. Evol. 34 (12), 3299–3302. doi: 10.1093/molbev/msx248 29029172

[B32] Slater G. S. Birney E. (2005). Automated Generation of Heuristics for Biological Sequence Comparison. BMC Bioinf. 6, 31. doi: 10.1186/1471-2105-6-31 PMC553969 15713233

[B33] Smith M. D. Wertheim J. O. Weaver S. Murrell B. Scheffler K. Kosakovsky Pond S. L. (2015). Less Is More: An Adaptive Branch-Site Random Effects Model for Efficient Detection of Episodic Diversifying Selection. Mol. Biol. Evol. 32 (5), 1342–1353. doi: 10.1093/molbev/msv022 25697341 PMC4408413

[B34] Stamatakis A. (2014). RAxML Version 8: A Tool for Phylogenetic Analysis and Post-Analysis of Large Phylogenies. Bioinformatics 30 (9), 1312–1313. doi: 10.1093/bioinformatics/btu033 24451623 PMC3998144

[B35] St John A. L. Rathore A. (2020). Early Insights Into Immune Responses During COVID-19. J. Immunol. 205 (3), 555–564. doi: 10.4049/jimmunol.2000526 32513850

[B36] Teng O. Chen S. T. Hsu T. L. Sia S. F. Cole S. Valkenburg S. A.. (2016). CLEC5A-Mediated Enhancement of the Inflammatory Response in Myeloid Cells Contributes to Influenza Virus Pathogenicity *In Vivo* . J. Virol. 91 (1), e01813–e01816. doi: 10.1128/JVI.01813-16 27795434 PMC5165214

[B37] Tillett R. L. Sevinsky J. R. Hartley P. D. Kerwin H. Crawford N. Gorzalski A.. (2021). Genomic Evidence for Reinfection With SARS-CoV-2: A Case Study. Lancet Infect. Dis. 21 (1), 52–58. doi: 10.1016/S1473-3099(20)30764-7 33058797 PMC7550103

[B38] To K. K. Hung I. F. Ip J. D. Chu A. W. Chan W. M. Tam A. R.. (2020). COVID-19 Re-Infection by a Phylogenetically Distinct SARS-Coronavirus-2 Strain Confirmed by Whole Genome Sequencing. Clin. Infect. Dis: an Off. Publ. Infect. Dis. Soc. America ciaa1275. doi: 10.1093/cid/ciaa1275 PMC7499500 32840608

[B39] Waterhouse A. Bertoni M. Bienert S. Studer G. Tauriello G. Gumienny R.. (2018). SWISS-MODEL: Homology Modelling of Protein Structures and Complexes. Nucleic Acids Res. 46 (W1), W296–W303. doi: 10.1093/nar/gky427 29788355 PMC6030848

[B40] Wilm A. Aw P. P. Bertrand D. Yeo G. H. Ong S. H. Wong C. H.. (2012). LoFreq: A Sequence-Quality Aware, Ultra-Sensitive Variant Caller for Uncovering Cell-Population Heterogeneity From High-Throughput Sequencing Datasets. Nucleic Acids Res. 40 (22), 11189–11201. doi: 10.1093/nar/gks918 23066108 PMC3526318

[B41] Wu J. T. Leung K. Leung G. M. (2020a). Nowcasting and Forecasting the Potential Domestic and International Spread of the 2019-Ncov Outbreak Originating in Wuhan, China: A Modelling Study. Lancet (London England) 395 (10225), 689–697. doi: 10.1016/S0140-6736(20)30260-9 PMC7159271 32014114

[B42] Wu J. Liu X. Liu J. Liao H. Long S. Zhou N.. (2020b). Coronavirus Disease 2019 Test Results After Clinical Recovery and Hospital Discharge Among Patients in China. JAMA Network Open 3 (5), e209759. doi: 10.1001/jamanetworkopen.2020.9759 32442288 PMC7244988

[B43] Wu Z. McGoogan J. M. (2020c). Characteristics of and Important Lessons From the Coronavirus Disease 2019 (COVID-19) Outbreak in China: Summary of a Report of 72 314 Cases From the Chinese Center for Disease Control and Prevention. JAMA 323 (13), 1239–1242. doi: 10.1001/jama.2020.2648 32091533

[B44] Xiao A. T. Tong Y. X. Zhang S. (2020c). False Negative of RT-PCR and Prolonged Nucleic Acid Conversion in COVID-19: Rather Than Recurrence. J. Med. Virol. 92 (10), 1755–1756. doi: 10.1002/jmv.25855 32270882 PMC7262304

[B45] Xie X. Zhong Z. Zhao W. Zheng C. Wang F. Liu J. (2020). Chest CT for Typical Coronavirus Disease 2019 (COVID-19) Pneumonia: Relationship to Negative RT-PCR Testing. Radiology 296 (2), E41–E45. doi: 10.1148/radiol.2020200343 32049601 PMC7233363

[B46] Yang Z. (2007). PAML 4: Phylogenetic Analysis by Maximum Likelihood. Mol. Biol. Evol. 24 (8), 1586–1591. doi: 10.1093/molbev/msm088 17483113

[B47] Ye X. Xiao X. Li B. Zhu W. Li Y. Wu J.. (2020). Low Humoral Immune Response and Ineffective Clearance of SARS-Cov-2 in a COVID-19 Patient With CLL During a 69-Day Follow-Up. Front. Oncol. 10, 1272. doi: 10.3389/fonc.2020.01272 32719750 PMC7348056

[B48] Yu Y. Li Y. Hu Y. Li B. Xu J. (2020). Breastfed 13 Month-Old Infant of a Mother With COVID-19 Pneumonia: A Case Report. Int. Breastfeeding J. 15 (1), 68. doi: 10.1186/s13006-020-00305-9 PMC7406867 32762723

[B49] Zehender G. Lai A. Bergna A. Meroni L. Riva A. Balotta C.. (2020). Genomic Characterization and Phylogenetic Analysis of SARS-COV-2 in Italy. J. Med. Virol. 92 (9), 1637–1640. doi: 10.1002/jmv.25794 32222993 PMC7228393

[B50] Zheng S. Fan J. Yu F. Feng B. Lou B. Zou Q.. (2020). Viral Load Dynamics and Disease Severity in Patients Infected With SARS-CoV-2 in Zhejiang Province, China, January-March 2020: Retrospective Cohort Study. BMJ 369, m1443. doi: 10.1136/bmj.m1443 32317267 PMC7190077

[B51] Zhou P. Yang X. L. Wang X. G. Hu B. Zhang L. Zhang W.. (2020). A Pneumonia Outbreak Associated With a New Coronavirus of Probable Bat Origin. Nature 579 (7798), 270–273. doi: 10.1038/s41586-020-2012-7 32015507 PMC7095418

[B52] Zhu N. Zhang D. Wang W. Li X. Yang B. Song J.. (2020). A Novel Coronavirus From Patients With Pneumonia in China 2019. N. Engl. J. Med. 382 (8), 727–733. doi: 10.1056/NEJMoa2001017 31978945 PMC7092803

